# The Gap Between the Perceptions and Expectations of Quality of Services Delivered to the Participants at the Scientific Conferences Using the University of Hafr Al Batin as a Model

**DOI:** 10.3389/fpsyg.2022.909489

**Published:** 2022-07-19

**Authors:** Abdullah Muzil Alharbi, Adel Ayed Alshammari, Hasnain Abbas Naqvi

**Affiliations:** ^1^Department of Education and Psychology, University of Hafr Al Batin, Hafar Al Batin, Saudi Arabia; ^2^Department of Management Sciences, University of Hafr Al Batin, Hafar Al Batin, Saudi Arabia

**Keywords:** scientific conferences, perception of quality services, expectation of quality services, service delivered, gap

## Abstract

This study aims to reveal the gap between the perceptions and expectations of the quality of services delivered to participants at scientific conferences held at emerging Saudi universities (using University of Hafr Al Batin as a model). The study also reveals if there are differences, with statistical significance, in the sample response due to several variants such as gender, nationality, and the attendance rate of conferences per year. The research adopts the descriptive approach and uses SERVQUAL instruments. It has been applied to a random sample of 155 persons. The study outcomes show that the expectations are higher than the perceptions in terms of all dimensions and also demonstrate the differences that exist due to the attendance rate of conferences per year in favor of those who attended three or more conferences. The study provides suggestions to raise the quality of services delivered to the participants at these conferences.

## Introduction

Scientific conferences have crucial objectives and goals that cannot be limited to only one aspect. Thus, universities exert huge efforts for the success of these conferences and to achieve the desired objectives and goals; otherwise, it could be a material and moral loss to the university. Julie (2005) refers that even scientific conferences have contributed to the national economy of the United Kingdom, with about 7.3 billion pound sterling in 2001, but several weak points affect the quality of services delivered in the conferences regarding facilities, tourist attractions, and many other factors, liable to harm the economic income.

The conference industry is one of the most dynamic developing service industries due to the increasing demand of individuals to exchange ideas and information (Shone, 2014). Therefore, giving attention to the competitive advantage that makes participants choose a conference among the other ones is very important. In this study, the importance of the quality of services delivered in conferences is highlighted as an essential competitive advantage for the success of the conferences and for ensuring effective participation.

Many studies have shown a low quality of services delivered in conferences reduces the level of effective participation, which all in all contributes to the success of the conferences and achieving their desired objectives. One of the studies referred to this is the Uzunboylu (2016) study that views the large differences in the expectations and perceptions of participants and the decrease in the level of satisfaction with provided services. Chuenjit (2019) study outcomes refer to the gap between the participants' perceptions and expectations about the quality of services delivered in conferences. Also, Reza et al. (2015) study show that the effect of the quality of location, quality access and journey to the conference, and the quality of the conference on the overall quality of services provided in it.

The emerging universities represent 66% of public universities, and despite that these universities have been established 15 or fewer years ago, they have held a large number of conferences in Saudi Arabia, but the delivered quality is not determined, as well as the extent of participants' satisfaction. This in turn reflects the extent of achieving its objectives and goals, in addition to its scientific reputation.

The University of Hafr Al Batin is considered one of the emerging universities whose establishment resolution has been issued in 2015. Despite being one of the recent universities, the University of Hafr Al Batin makes sure of holding international conferences that bring together a large number of participants. Thus, it seeks to develop its academic community, as well as local and student communities. Both Abdel-Gawad and Al-Khatib (2013) have referred to that one of the emerging universities' mechanisms is conducting seminars, workshops, and conferences.

Considering the huge importance of scientific conferences and their desired objectives, their success is highly contributed to the level of participation, the value of scientific articles discussed in it, and no doubt this is related to the scientific and the academic level of participants themselves and their nationalities. They decide whether to participate or not in the periodic conferences based on several criteria, and the most important one is the content of the conference.

Perhaps the gap analysis between the quality of delivered services and that expected by participants can provide a vision needed to decide in respect of conducting conferences, thus increasing its chances for success and achieving its goals. This study is one among many that focuses on viewing the quality of services delivered in the scientific conferences held at the emerging universities with application to the University of Hafr Al Batin.

## Theoretical Framework

### Quality of Service

Some studies indicate that quality of service is one of the concepts that “connect to the ability of organizations to meet the desires of beneficiaries and achieve satisfaction for them, and also concepts related to the ability of these organizations to support their presence in a competitive manner, which made the quality of service gain importance in administrative and educational thought” (Ezz El Din, 2016, p. 83).

The concept also from the methodological point is considered one of the component concepts as it includes “a set of dimensions that make up its conceptual content as they are considered criteria used by customers to judge the quality of service or that represent their perceptions of it” (Al-Ziyadat and Al-Alwan, 2020, p. 161).

Several studies also agree that the concept of quality of service is reflected in the service beneficiary's evaluation of the degree of excellence or overall superiority in the performance of the service, or by fulfilling the requirements of customers, or the result of the difference between the final expectations of the beneficiaries for excellent service and their awareness of the performance of the service they receive. This, of course, led to making the issue of criteria for measuring and presenting them characterized by lack of clarity and discrepancy, and then disagreement about the methods of measuring quality and performance indicators (Al-Awlaki, 2018). Therefore, the issue of measuring the quality of service in many fields and various activities is one of the topics that have dominated social and educational thought in general in recent times, especially among those working in the field of administrative and educational sciences.

The service of university higher education activities refers to “the state of the service in general, which has a set of characteristics, including it is intangible, and has direct contact with the beneficiaries. It is also not possible that it is not stored, but rather presented in an instant form, and its owners cannot be transferred or resold, and it cannot be transferred from one place to another” (Jibreen, 2006).

Given the specificity of educational service activities and the presence of the beneficiary of them face to face in many situations with service providers in time and place, this situation is necessarily affected by several factors such as the appearance of both the interior and the exterior design of the educational service places, their characteristics, extracurricular activities, and the tools and equipment used for entertainment, places to provide educational service, and others (Zeithaml et al., 2006).

Perhaps the first serious attempt to measure service is attributed to Parasuraman and his colleagues, in their attempt to design an initial scale consisting of 10 dimensions (Parasuraman et al., 1985). Then they were able to avoid many of the defects of the previous scale and designed the famous (SERVQUAL) scale, which consists of five dimensions (Parasuraman et al., 1988), also known as the gap model and as the gap scale, and its validity test to measure what is known as the five gaps of service quality. Carlett and his colleagues indicate that service is measured (Carrillat et al., 2007)—usually—in five main dimensions:

*Tangible*: It means all the tangible components of the institution that provides the service, such as the material facilities associated with providing the service, such as the physical facilities, equipment, and the personal appearance of the employees.

*Reliability*: It means depending on the institution in its ability to perform the promised service reliably and accurately.

*Responsiveness:* It means the institution workers' readiness to help the beneficiaries, providing services when ordered, and responding to inquiries on time.

*Assurance:* It means the workers' ability to instill confidence and feeling of acceptance and provide safety and security in customers' souls.

*Empathy:* It means the care and interest provided by the institution toward customers to feel that they are their central concern and that their interests are essential.

### Literature Review

The most topic-related studies are selected, presented in a historical sequence of their proceeding from the oldest to the newest, as follows:

A descriptive study conducted by Chiu and Ananzeh (2012) to identify the conference role in the tourism industry in Jordan. The results revealed that the factors affecting the decision of participation involve the affordability and availability of transportation, security, safety, and quality of services. Moreover, there are differences among the domestic and international participants in favor of the domestic participants, in addition to the differences between female and male participants in favor of female participants. There are also differences between local and international participants in favor of locals, and between female and male participants in favor of female participants.

Sababhi (2015) conducted a study aimed at analyzing the structure of the business tourism environment in the Kingdom of Saudi Arabia and used the descriptive–analytical method and SWOT analysis. Among its results were the business tourism environment in the Kingdom of Saudi Arabia is attractive and enjoys a global reputation based on the political, economic, and religious power of the Kingdom, but these advantages have not been exploited in making Saudi Arabia a center for business, tourism, and conferences in the region, and that currently held in Saudi Arabia currently is not appropriate with its global reputation.

Uzunboylu (2016) conducted a study to evaluate the quality of service at the international educational conference and used the descriptive method with the Servqual scale. The results show that there is a great difference between participants' expectations and perceptions and also revealed a low level of satisfaction with the fact that the quality of the service was a dynamic process that required continuous improvement. Past experiences of participants may give the key overview of their expectations as to what the future level of conference services should be.

In their study, Mekkawy et al. (2017) aimed to identify the reality of tourism conferences in Egypt and to propose a strategy for its development and it used the descriptive method. The results show that the most important requirement for the organization of tourism conferences is the need for equipped conference rooms with adequate audio-visual facilities, providing easy and accessible means of transportation and reducing the costs of accommodation and services.

Chuenjit (2019) examined customer satisfaction toward service quality and communications for the Power Pool, Conference in Bangkok, where the study included a sample of 400 individuals, and the results of the study showed that there is a gap between the participants' expectations and perceptions in all dimensions, the largest of which was in the reliability dimension, then the response, followed by the sympathy. This difference led to demographic factors of the respondents and recommended the need to improve the reliability of conferences through teamwork and effective communication using modern technology and social media.

Zeljko et al. (2019) conducted a study titled “new hybrid model” for assessing the quality of scientific conferences based on BWM Rough and SERVQUAL, and one of its most important objectives was to identify signatures and notes to evaluate the quality of Horizons New Conference, which was held on November 17–18, 2017, in Doboj. The study showed high expectations of participants, as well as a higher average in the observations, indicating the satisfaction of the participants with the general organization of the conference.

Finally, Cassar et al. (2020) conducted a study to identify the factors affecting attendance at conferences and used a descriptive method. The results show that quality of service, time, reasonable cost, and quick response to participant service have an influence on the decision to participate, while recreational facilities and luxury hotel accommodation did not play a major role in motivating attendance.

Despite the importance of scientific conferences held at universities, the small number of studies that try to view the quality of its delivered services—according to researchers' outcomes—which reflect negative results (Uzunboylu, 2016; Chuenjit, 2019). The literature refers to the obvious lack of research addressing the activities of such conferences and their delivered services in the Arab nation, especially Saudi Arabia, even with the great position of these conferences in academia and among those concerned with scientific research (Rowe, 2019), in addition to raising the competitiveness of Saudi universities (Al-Abbad, 2017).

When the delivered services at scientific conferences have become one of the competitive advantages of the conference, and after many emerging Saudi universities have started to be concerned with those conferences, the need for assessing the quality of its services has arisen, in addition to the creation of an environment that could attract specialists and scientifically concerned people from different nationalities to achieve the scientific goals of the conference and gain a good reputation.

If the success of scientific conferences highly depends on the type of participants, their scientific position, and multiple nationalities, the level of their satisfaction about the delivered services is an important means that lead to the success of the given conference. Hence, this study aims at viewing and examining the quality of delivered services at the Saudi emerging universities by measuring the quality of the scientific conference held recently at the University of Hafr Al Batin under the title of the First International Conference on Applied sport psychology “reality and ambition”, providing a clear answer to whether there is a gap between the expectations and perceptions regarding this conference which falls under the real gap, and also by using the SERZQUAL scale and providing a clear vision about this reality, as well as providing suggestion to help enhance the quality of services.

The concept of SERVQUAL is based on the gap theory, which states that the expectations and perceptions of customers are the factors that influence the quality of service. This is a multiple-item scale that can be used to measure the service quality. In their subsequent work, Berry, Parasuraman, and Zeithaml proposed a set of 10 quality dimensions that can be used to evaluate the service quality of a company. These include responsiveness, reliability, security, access, credibility, and understanding the customers. Due to the complexity of the service quality measurement process, the original 10 dimensions were then reduced to five dimensions.

The SERVQUAL questionnaire features 22 pairs of questions that are designed to capture various dimensions of service such as reliability, trustworthiness, and empathy. The first 22 statements are designed to capture the customers' expectations, while the corresponding statements are used to reflect the perceptions of the customers regarding the service quality of an organization (Fick and Ritchie, 1991). This method can also help customers identify areas of improvement and how they can improve the service they receive. In addition, it can help them describe the organization's operations and how they have been operated to their satisfaction (Langer, 1997).

## Methodology

The research used the descriptive survey method, which is the most reliable method for understanding and analyzing various aspects of the phenomenon.

The study attempted to answer the following questions:

- Are there any differences with statistical significance between perceptions and expectations of participants regarding the level of quality dimensions of the service delivered at the scientific conference held at the University of Hafr Al Batin?
- Are there any differences with statistical significance between perceptions and expectations of participants regarding the level of quality dimensions of the service delivered at the scientific conference held at the University of Hafr Al Batin and those attributable to the following variants (gender, nationality, and attendance rate of conferences per year)?
- What are the suggestions for raising the quality of service delivered at the scientific conference held at emerging Saudi universities?

### Research Sample

The original community, which includes participants in the First International Conference on Applied Sports Psychology “Reality and Ambition,” organized by the University of Hafr Al Batin, consists of a total of 680 conference participants. A random sample of 155 participants was selected, representing 22.79% of the original community. The measure was distributed in two phases: the first was at the beginning of the first day of the conference and dealt with what the participants expected from the services, and as for the second phase, it was at the end of the last day of the conference and dealt with the services that the participants already realize. The characteristics of the research sample members can be explained in Table 1 as follows:

**Table 1:** Number of copies of the measure distributed and retrieved.

**Participation in** **conferences** **annually**	**Gender**	**Nationality**							
		**Saudi**				**Saudi**			
		**First round**		**First round**		**First round**		**First round**	
		**Distributed**	**Distributed**	**Distributed**	**Distributed**	**Distributed**	**Distributed**	**Distributed**	**Distributed**
3 at least	Male	20	18	20	19	12	9	12	8
	Female	59	56	59	57	15	12	15	13
More than 3	Male	22	18	22	19	13	8	13	10
	Female	8	5	8	7	6	4	6	5
Total		109	97	109	102	46	33	46	36
Distributed in the first round		155	Retrieved in the first round					130	83.90%
Distributed in the second round		155	Retrieved in the second round					138	89.00%

Given the difference in return in the first round from the second, and the need for statistical analysis and comparison to equal the number of the two phases, eight copies of the return were deleted in the second round, chosen at random so that the number of each round became 130.

### Tool and Its Codification

The research used the SERVQUAL scale to measure the gap between perceptions and expectations in the quality of service provided (Parasuraman et al., 1988), which consists of 22 statements divided into five dimensions: tangibility (4) statements, reliability (5) statements, responsiveness (4) statements, assurance (4) statements, and empathy (5) statements. The researcher has translated the measure and formulated its statements to be compatible with the study community while preserving its dimensions and content.

#### Validity and Reliable

The researcher verified the validity and reliability of the measure through the following procedures:

##### Face Validity

The tool was presented in its initial form, consisting of 22 statements in five dimensions for 12 teachers of education to explore their opinions and benefit from their observations about the tool items in terms of formulation, suitability for the field, and the degree of achieving the goal for which it was set. Statements with <90% agreement of the arbitrators were deleted, and some expressions were modified in light of the comments made by the arbitrators. Then, the form was reformulated to its final form, which consisted of 20 statements represented by 4 statements for each of the five dimensions.

##### Internal Consistency Validity

Internal consistency validity was calculated by finding the correlation coefficient between each of the statements of the dimension and the total degree of the dimension to which the statement belongs, that is, after applying the tool to an exploratory sample, and the results showed a high degree of correlation, which confirms the internal consistency validity of the tool, as shown in Table 2.

**Table 2:** Correlation coefficients for each dimension statement with the total degree of the dimension to which it belongs.

**Dimensions**	**Correlation coefficient**	**Dimensions**	**Correlation coefficient**	**Dimensions**	**Correlation coefficient**
Tangibility		Reliability		Empathy	
1	^**^0.766	1	^**^0.604	1	^**^0.719
2	^**^0.741	2	^**^0.604	2	^**^0.719
3	^**^0.603	3	^**^0.793	3	^**^0.775
4	^**^0.806	4	^**^0.582	4	^**^0.693
Responsiveness dimension		Assurance dimension			
1	^**^0.640	1	^**^0.679		
2	^**^0.640	2	^**^0.679		
3	^**^0.841	3	^**^0.807		
4	^**^0.617	4	^**^0.654		

** *Function at 0.01 level*.

#### Stability of the Research Tool

Tool stability was calculated using Cronbach's alpha, and Table 3 shows the value of the stability coefficient for each part of the measure.

**Table 3:** Values of stability coefficients for each of the measure dimensions.

**Dimensions**	**Stability coefficient**	**Dimensions**	**Stability coefficient**
Tangibility	0.791	Assurance	0.832
Reliability	0.863	Empathy	0.846
Responsiveness	0.852		
Overall Resolution		0.835	

The previous table shows that the values of the stability coefficients are high, which indicates that the measure enjoys a high degree of stability, and therefore, the tool used is characterized by validity and reliability and can be used scientifically.

#### Methods of Statistical Processing

A three-point Likert scale was applied, where scores of 1, 2, and 3 were given for the responses agree, somewhat agree, and disagree, respectively, as well as the statistical methods that meet the nature of the study to verify its hypotheses using the Statistical Package for Social Sciences (SPSS).

##### Relative Weight

The relative weight, which ranges between 1 and 1.66 refers to a low estimation, a relative weight that ranges between 1.67 and 2.33 refers to a medium estimation, and the relative weight of 2.34–3 refers to a high estimation, and it should be noted that the period used here is 3/2, which is ~0.77. The judging criteria over the values of the relative weights were calculated according to the following equation: The highest degree—the lowest degree on the response periods.

## Results and Its Discussion

a. *Results related to the first question:*

Are there statistically significant differences between participants' perceptions and expectations on the level of quality dimensions of services provided at the scientific conference held at the University of Hafr Al Batin?

To answer this question, we calculated the arithmetic averages of participants' perceptions and expectations on the level of quality dimensions of services provided at the conference and the phrases they contain. If the expected quality is greater than the perceived service, the quality of the service provided will be less than satisfactory, and if the expected quality is equal to the perceived quality of service, then it will be satisfactory. In addition, if the expected quality is less than the perceived quality, the quality of the service provided will be more than satisfactory, and it tends toward the optimum quality as the gap between them was calculated using the *t*-test, as shown in Table 4.

**Table 4:** Participants' perceptions and expectations for each of the dimensions of the provided service, the gap between them, and the calculated *t*-value.

**Sr. No**.	**Phrases**	**Average of the** **expected service**	**Average of the** **perceived service**	**The gap**	**(*T*) Value**
**First: tangibility dimension**					
1	The service facilities and utilities needed by the participants are available.	2.48	1.58	0.89	^**^–10.189
2	Service providers are giving attention to their appearance.	2.55	1.91	0.64	^*^–7.392
3	The conference location is easily accessible.	2.64	2.65	−0.01	0.227
4	The view of the buildings creates a comfortable educational atmosphere.	2.65	1.77	0.88	^**^–10.055
	**The average of the tangibility dimension**.	**2.57**	**1.97**	**0.60**	^**^–**10.399**
**Second: assurance dimension**					
5	Services are performed on time without delay.	2.54	2.24	0.31	^*^–5.478
6	The conference researches have a practical importance.	2.81	2.33	0.47	^**^–10.648
7	The behavior of the organizers gives an impression of confidence to the participants.	2.71	2.09	0.62	^**^–11.220
8	The organizers provide an accurate and true information of the events.	2.56	2.08	0.48	^*^–7.942
	**The average of the assurance dimension**.	**2.66**	**2.27**	**0.39**	–**10.340**
**Third: responsiveness dimension**					
9	The organizers of the conference are keen to automate the administrative work of the conference.	2.78	2.23	0.56	^*^–9.306
10	Service providers respond to the needs of the participants.	2.76	1.68	1.08	^**^–18.227
11	There are enough providers for the needed Service of the participants.	2.74	1.66	1.08	^**^–17.083
12	The organizers concern about solving the problems of the participants in a fully transparent manner.	2.68	2.69	−0.01	1.196
	**The average of the responsiveness dimension**.	**2.69**	**2.06**	**0.64**	^**^–**16.830**
**Forth: reliability dimension**					
13	The discussion of the researches are made in a democratic atmosphere.	2.74	2.02	0.73	^**^–12.727
14	The organizers have high skills in providing the service.	2.59	2.18	0.41	^**^–7.491
15	The organizers have a great credibility in dealing with others.	2.69	2	0.7	^**^–11.572
16	safety regulations are available in the location, where the conference will be held.	2.49	1.82	0.67	^**^–13.332
	**The average of the reliability dimension**.	**2.67**	**2.06**	**0.61**	^**^–**13.332**
**Fifth: empathy dimension**					
17	The service providers are giving attention to fitness.	2.77	2.46	0.31	^*^–5.620
18	Secduale of conference‘s program are appropriate to the participants.	2.73	1.81	0.92	^**^–13.150
19	There will be methods of acquaintance in an atmosphere of familiarity during the sessions and seminars.	2.74	2.10	0.64	^**^–11.040
20	The organizers are putting the benefit of the participants in front of their concerns.	2.77	1.85	0.91	^**^–15.310
	**The average of the empathy dimension**.	**2.77**	**2.14**	**0.63**	^**^–**16.350**
	**Total degree of the questionnaire**	**2.68**	**2.11**	**0.570**	^**^–**14.39**

*>> More than 0.05 is insignificance.*

* *If it reached 0.05, it will be an average significance;*

** *If it was 0.01, it will be a high significance*.

Table 4 shows that the following:

- The arithmetic averages of all the expected quality dimensions reached (2.68), which is located in the high estimation level according to the relative weight adopted for the interpretation of the results.
- The arithmetic averages of all the perceived quality dimensions reached 2.11, which is located in the medium estimation level according to the relative weight adopted for the interpretation of the results. This result demonstrated that the expectations of participants are higher than their perceptions, which means that the quality of provided service was less than satisfactory. The reason for this may be due to the ambitions, which always overcome individuals, and that their expectations may be much higher than their perceptions, especially when the service is related to scientific aspects, the participants voluntarily attend. This is also due to the conferences being held in a country, which is at the head of universities world ranking; this made the participants have high expectations for the service. This result is consistent with that of Uzunboylu (2016) and Chuenjit (2019), while it differs from the result of the study of Zeljko et al. (2019).
- The statistical difference between the expected quality and the perceived quality regarding all dimensions is 0.57, which is indicative of 0.01 significance level. The largest value of the gap was in responsiveness and amounted to 0.64, while the smallest gap was in reliability and amounted to 0.39.
- Empathy ranked first under the quality of expected service, with an average of 2.77. This indicates the level of participants' ambitions and suggestions they have regarding the received services.
- Reliability ranked first under the quality of the perceived service, with an average of 2.27. This indicates what the participants experienced during the actual performance and the extent of their conviction.
- The largest gap was in the sentence “The service providers respond to the needs of participants” and “There is an adequate number of service providers who deliver services required by participants”—both represent the highest positive gap regarding responsiveness, with an average of 1.08. Maybe the reason behind this large gap is the novelty of conducting scientific conferences at the university.
- The smallest gap was in the sentence “Conference organizers make sure to solve the participant problems”; the value of the gap is negative in favor of the perceptions, with an average of −0.01. This is more than a satisfying value, which is leveled up to almost the perfect quality. The reason behind this is that the conference organizers think of this as the best way to enhance trust and relationship between them and the participants.
- The highest value of positive gap in empathy was in “the timing of conference program suits all participants,” with an average of 0.92. Many participants think of the inappropriateness of the timing of the conference. Therefore, many of them apologize for not attending. Thus, the organizing body has to set the date of the conference precisely in the head of time as a large number of apologies are enough to postpone the conference. The outcomes of Cassar et al. (2020) study reveal that the date/time of the conference is from the encouraging factors for attending the conference. While the least value of the positive gap was in “service provider's concern about their courtesy and the propriety of the organization,” with an average of 0.31. This is due to the lack of oversight on the service providers by the organizers.
- Assurance ranked third, with a gap value of 0.61, where the sentence “research studies are discussed in democratic environment” records the highest value regarding this dimension, with an average of 0.73. Participants think that discussions develop the research scientific skills, engage them with those interested in their field of research, and in addition, enable them all to identify strengths and weaknesses of research discussed at the conference. The least positive value of the gap was in “Organizers have high skills in terms of provision of service,” with an average of 0.41.
- Tangible ranked fourth, with a gap value of 0.60. “All service facilities needed are available for participants” records the highest positive value regarding this dimension, with an average of 0.89. This is due to the non-availability of proper places designed to conduct the conference and the large distance between the facilities and the difficulty in moving between them, which will result in consuming effort and time to move to and from the place of conference. This is similar to the outcomes of Hinkin and Tracey (2003), study which provide the importance of giving attention to the supplies and services provided during the conference. Conference organizers have to consider the quality of facilities and supplies upon choosing the place for a conference. The sentence “Service providers care about how they look” represents the least negative value of the gap, with an average of −0.01. Therefore, the quality of service is more than the satisfying value, which is leveled up to almost the perfect quality. This is due to the organizational body's concern about their look, believing that this will reflect on the reputation of the entity, thus gaining new clients.
- Reliability ranked fifth and last, with a gap value of 0.39. “Organizers' behavior gives the impression of confidence to participants” represents the highest positive gap in this dimension, with an average of 0.62, the reason behind this being the organizer's behavior does not fulfill the participants' needs, while “Services are delivered in due time without delay” represents the least positive value, with an average of 0.31. Organizers have to priorly coordinate with their employees. This result differed from the results of Chuenjit (2019) study which showed the largest gap in the reliability dimension, while it is similar to the results of the Uzunboylu (2016) study, which hopes from regulators more in terms of time management about the services provided.
- The outcome of this study coincides with Uzunboylu (2016) study regarding the existence of a gap between the perceptions and expectations, which reduces the level of participants' satisfaction. In addition, the study also coincides with the outcomes of Sababhi (2015) study in the aspect that the currently held conference correspond neither with the international reputation nor with the advantages of the Kingdom of Saudi Arabia. Mentioning the outcomes of Mekkawy et al. (2017), all these studies together, with this one, are in agreement that all events. Conferences held in the Republic of Egypt do not correspond to the level of advantages owned. Finally, the previous leading and international expertise should be utilized to have the industry conducting scientific conferences.

b. *Results related to the second question:*

Are there statistically significant differences between the participants' perceptions and expectations for the level of dimensions of quality of service provided in the scientific conference held at the University of Hafr Al Batin that are attributed to the following variables (gender, nationality, and annual conference participation rate)?

To answer this question, a *t*-test is used to identify the statistically significant differences according to the study variables. The results are given in Table 5.

**Table 5:** *T*-test shows differences in the sample individuals' responses according to the gender variable by calculating the gap.

**S.N**	**Dimensions**	**Social gender**	**No**.	**Arithmetic** **average**	**Standard** **deviation**	**Degree of** **freedom**	***T*-value**	**Statistical** **significance**
1	Responsiveness	Male	53	0.65	0.40	128	1.378	^*^0.089
		Female	77	0.60	0.37			
2	Empathy	Male	53	0.61	0.38	128	0.718	^*^0.302
		Female	77	0.65	0.40			
3	Assurance	Male	53	0.62	0.38	128	0.663	^*^0.278
		Female	77	0.58	0.36			
4	Tangibility	Male	53	0.57	0.35	128	0.838	^*^0.352
		Female	77	0.62	0.38			
5	Reliability	Male	53	0.37	0.23	128	0.469	^*^0.197
		Female	77	0.40	0.25			
6	Overall resolution	Male	53	0.59	0.37	128	0.813	^*^0.342
		Female	77	0.54	0.33			

* *Significance level is more than 0.05*.

Table 5 shows that there are no significant differences between the participants' perceptions and expectations for the level of dimensions of quality of service provided in the conference that are attributed to the gender variable. Perhaps, this is because the service provided to male and female participants has no difference in form or content. Service is measured through its function. Moreover, the participants concentrate on the effectiveness of the service and its suitability to their needs more than biological division, in which differences between the two genders dissolve.

This result differs from Chiu and Ananzeh (2012) study results which reveal that there are differences in the factors affecting the decision-making of participation in conferences between female and male participants in favor of female participants.

As for the differences between sample individuals' responses according to the nationality variable are in Table 6.

**Table 6:** *T*-test shows differences in the sample individuals' responses according to the nationality variable by calculating the gap.

**S.N**	**Dimensions**	**Nationality**	**No**.	**Arithmetic** **average**	**Standard** **deviation**	**Degree of** **freedom**	***T*-value**	**Statistical** **significance**
1	Responsiveness	Saudi	97	0.67	0.41	128	0.923	^*^0.104
		Non-Saudi	33	0.58	0.37			
2	Empathy	Saudi	97	0.63	0.38	128	0.625	^*^0.353
		Non-Saudi	33	0.63	0.4			
3	Assurance	Saudi	97	0.64	0.39	128	0.577	^*^0.325
		Non-Saudi	33	0.56	0.36			
4	Tangibility	Saudi	97	0.59	0.36	128	0.729	^*^0.412
		Non-Saudi	33	0.6	0.38			
5	Reliability	Saudi	97	0.37	0.23	128	0.408	^*^0.230
		Non-Saudi	33	0.4	0.25			
6	Overall resolution	Saudi	97	0.61	0.37	128	0.707	^*^0.400
		Non-Saudi	33	0.52	0.33			

* *Significance level is more than 0.05*.

Table 6 shows that there are no significant differences between the participants' perceptions and expectations for the level of dimensions of quality of service provided in the conference that are attributed to the nationality variable. Perhaps, this is because there is no discrimination between participants regarding their nationalities; all their participations are free.

The statistical differences according to the conference participation rate variable are given in Table 7.

**Table 7:** *T*-test shows differences in the sample individuals' responses according to the annual conference participation rate variable by calculating the gap.

**S.N**	**Dimensions**	**Annual** **conference** **participation rate**	**No**.	**Arithmetic** **average**	**Standard** **deviation**	**Degree of** **freedom**	***T*-value**	**Statistical** **significance**
1	Responsiveness	3 at least	59	0.5	0.41	128	10.771	0.007^**^
		More than 3	35	0.73	0.37			
2	Empathy	3 at least	59	0.47	0.38	128	7.294	0.025^*^
		More than 3	35	0.69	0.4			
3	Assurance	3 at least	59	0.48	0.39	128	6.734	0.023^*^
		More than 3	35	0.62	0.36			
4	Tangibility	3 at least	59	0.44	0.36	128	8.507	0.029^*^
		More than 3	35	0.66	0.38			
5	Reliability	3 at least	59	0.28	0.23	128	5.577	0.016^**^
		More than 3	35	0.44	0.25			
6	Overall Resolution	3 at least	59	0.46	0.37	128	8.251	0.028^*^
		More than 3	35	0.57	0.33			

* *A function at 0.05 of medium significance;*

** *A function of 0.01 of high significance*.

Table 7 shows that there are fundamental differences between participants' perceptions and expectations of the level of service quality dimensions provided in the conference due to the variable rate of attendance at conferences per year in favor of those who attended more than three conferences. This may be due to the previous experiences of the participants and the number of experiences they gained as a result; the more their participation in previous conferences, the more likely they are to evaluate this conference based on their previous experiences. Evaluating the participants' past experiences may give the main insights into their expectations about what the level of services for future conferences should be. This result is consistent with the results of Uzunboylu (2016) study in that the participants' past experiences give the main insights into their expectations about what the level of conference services should be in future.

c. *Results related to the third question:*

What are the proposals to raise the quality of service provided in scientific conferences in emerging universities?

In light of the results of the study, a set of proposals can be presented that can raise the quality of service provided to participants in the scientific conference at the University of Hafr Al Batin in particular and emerging Saudi universities in general, according to the SERVQUAL scale, as follows:

- *Tangibility dimension*
  - Working on providing service facilities and utilities needed by the participants, such as halls that meet the expected number of participants in a healthy manner and suitable condition.
- *Assurance dimension*
  - Showing appreciation to the participants and making them aware of the importance of the research that they submit.
  - Providing accurate and correct information about the conference and its activities.
- *Responsiveness dimension*
  - The management of the conference is keen to provide the needs of participants and attendees quickly and accurately and showing transparency in determining the date of obtaining and completing the service.
  - Providing a sufficient number of service providers trained in organizing and managing conferences and serving the participants.
  - Selecting the employees who have a complete willingness and desire to provide the necessary and immediate assistance to the participants.
- *Reliability dimension*
  - Providing the element of transparency and academic democratic atmosphere in the presentations, discussions, and seminars of the conference and giving participants the freedom to express their opinions without hesitation.
  - Providing the organizational confidence for attendees and participants to make a cooperative atmosphere in the conference.
  - Providing working staff with sufficient knowledge to answer the inquiries and questions of the participants and attendees.
- *Empathy dimension*
  - Making sure that the schedule of the conference is appropriate and suits the largest possible number of participants.
  - Enhancing the human relations between the conference management and the organizers, on the one hand, and the participants, on the other hand, during breaks and ensuring the exchange of experiences and advice.
  - Giving due consideration to the interests of the participants and attendees, listening to their opinions and suggestions and not neglecting them.

## Conclusion

The findings of the current study are given in the following text:

- The conference participants' perceptions of the level of service quality dimensions were lower than their expectations.
- There are statistically significant differences between participants' perceptions and expectations of the level of service quality dimensions provided in the conference.
- There are no statistically significant differences between the participants' perceptions and expectations of the level of service quality dimensions provided in the conference due to the two variables (gender and nationality).
- There are statistically significant differences between participants' perceptions and expectations of the level of service quality dimensions provided in the conference due to the variable rate of attendance at conferences year in favor of those who attended three conferences or more.
- Such conclusions may be applied to the provided services in conferences held at emerging Saudi universities, although they may vary if we generalize them to the universities outside the Kingdom.

## Recommendations

Based on the results and conclusions of the study, we recommend the following:

- Paying more attention to the tangibility dimension, which had the lowest perceived average gap, and the responsiveness dimension, which showed the largest gap between the expected and the perceived service.
- Carrying out similar studies in old universities and making comparisons between their results.
- Taking advantage of the local, regional, and international experiences regarding the same.
- Promoting and enhancing the level of provided services in the conferences through employment.

## Data Availability Statement

The raw data supporting the conclusions of this article will be made available by the authors, without undue reservation.

## Ethics Statement

Ethical review and approval was not required for the study on human participants in accordance with the local legislation and institutional requirements. Written informed consent from the participants was not required to participate in this study in accordance with the national legislation and the institutional requirements.

## Author Contributions

AMA: main idea and review of literature. AAA: methodology and analysis. HN: data collection. All authors contributed to the article and approved the submitted version.

## Conflict of Interest

The authors declare that the research was conducted in the absence of any commercial or financial relationships that could be construed as a potential conflict of interest.

## Publisher's Note

All claims expressed in this article are solely those of the authors and do not necessarily represent those of their affiliated organizations, or those of the publisher, the editors and the reviewers. Any product that may be evaluated in this article, or claim that may be made by its manufacturer, is not guaranteed or endorsed by the publisher.
